# One world, one goal: Advocacy and policy for a unified Parkinson's response

**DOI:** 10.1177/1877718X261448383

**Published:** 2026-05-07

**Authors:** Natasha Fothergill-Misbah

**Affiliations:** 1 Population Health Sciences Institute, Faculty of Medical Sciences, Newcastle University, Newcastle upon Tyne, UK

**Keywords:** neurological disorders, brain health, health equity, global health governance

## Abstract

Parkinson's disease is one of the fastest growing neurological disorders with regards to disability and death. Though this burden is felt globally, inequities in research, advocacy, prioritization and funding mean that the needs of people with Parkinson's disease in low- and middle-income countries, and from marginalized communities in high-income countries, remain poorly recognized. Parkinson's disease is increasingly being acknowledged as a global public health issue requiring a public health response. Global advocacy efforts, awareness-raising initiatives, research collaborations, partnerships and investment in Parkinson's disease have therefore accelerated in recent years, with the positioning of people affected by PD as authoritative voices paramount to this drive. Yet despite this progress, inequalities in access to treatments, care and support persist. Responding to the global burden posed by Parkinson's disease requires sustained, multi-sectoral, concerted action, building on an integrated and systems-oriented approach. The generation of momentum for a public health approach to Parkinson's disease must be met with implementation at the country level, alongside sufficient allocation of resources, monitoring and evaluation. The approach offered in this paper builds on the five cross-cutting strategic objectives outlined in the World Health Organization's Intersectoral global action plan on epilepsy and other neurological disorders 2022–2031 (IGAP) implementation toolkit, offering a platform to build a unified global response to Parkinson's disease. Action should therefore center on the integrated themes of (1) prioritization and governance; (2) diagnosis, treatment and care; (3) promotion and prevention; (4) research and information systems; and (5) a tailored Parkinson's disease-specific public health approach.

## Plain language summary

A global response to Parkinson's disease

Parkinson's disease is one of the world's fastest growing brain disorders. While Parkinson's disease affects people across the globe, individuals living in low- and middle-income countries, and those from marginalized communities in higher income countries, do not receive the attention, care and support they need to live well. Parkinson's disease has been recognized as an important global public health issue, including by the World Health Organization. As a result, international advocacy efforts, awareness campaigns, research efforts and investments have grown. People with lived experience are also increasingly involved in shaping these efforts, which is an important step forward. Despite this progress, there are still inequalities in who can access a diagnosis, treatment and care. Addressing the challenge of Parkinson's disease requires long-term and coordinated action across many sectors. Efforts need to be integrated into broader health systems and supported by strong political commitment. The recognition of Parkinson's disease as a public health priority must be met by plans to implement strategies, accompanied by monitoring to understand if the strategies are working. This paper offers recommendations that build on the World Health Organization's approach to tackling neurological disorders through an ‘intersectoral global action plan’. These objectives provide a foundation for building a unified global response to Parkinson's disease. The key areas for action include: (1) stronger prioritization and leadership; (2) improved access to diagnosis, effective treatment and care; (3) the promotion of brain health and disease prevention; (4) improved research and health information systems; and (5) tailored public health strategies specific to Parkinson's disease. Together, these actions will help to reduce inequalities and ensure that all people impacted by Parkinson's disease can receive the care and support they need, no matter where they live.

## The case for a unified global Parkinson's disease response

Parkinson's disease (PD) has been determined as one of the fastest growing neurological disorders with regards to disability and death.^1^ The Global Burden of Disease (GBD) study estimated 11.8 million people living with PD in 2021, an increase of 274% from 1990.^1,2^ The age-standardized global prevalence of PD in 2021 was 138.6 per 100,000 people (168.2 in males and 114.5 in females),^1^ with a continuous projected upward trend in burden. However, the true prevalence remains challenging to determine,^2–5^ largely due to reporting biases, inconsistencies in data collection and reporting, and the under-diagnosis of PD in low- and middle-income countries (LMICs) and among under-represented groups in high-income countries (HICs).^6–8^ Almost half of the global population of persons living with PD reside in LMICs,^9^ yet research, advocacy and funding remain centered in HICs.

The significant burden posed by PD to individuals, families and society – physically, socially and economically – reflects the recognition of PD as a global public health issue requiring a unified public health response.^10^ To outline and address these gaps and needs, the World Health Organization (WHO) published a ‘Technical Brief’ on PD in 2022. The brief outlined key actions to develop strategies, programs and services to improve the lives of persons impacted by PD, forming the ‘Six Action Steps’ to address global disparities in PD:^11^ disease burden; advocacy and awareness; prevention and risk reduction; diagnosis, treatment and care; caregiver support; and research.

The subsequent inclusion of PD as a ‘tracer condition’ in the WHO's ‘Intersectoral global action plan on epilepsy and other neurological disorders 2022–2031’ (IGAP) is an acknowledgement of its growing burden.^12^ Tracer conditions are carefully chosen diseases used to shine a light on systemic problems that affect a wider group of conditions. In this case, PD (among other conditions) was identified by WHO to understand barriers that affect many other neurological disorders. IGAP – a 10-year action plan – was adopted by all countries at the 75^th^ World Health Assembly to promote and support a comprehensive, coordinated response to neurological disorders across multiple sectors. The goal of IGAP is to reduce the stigma, impact and burden of neurological disorders (including PD) and to improve quality of life for people with neurological disorders, their carers and families. WHO has published an IGAP ‘toolkit’ to provide resources for countries in the implementation of IGAP.^13^ This is intended as a ‘go-to’ resource for stakeholders to approach country-level, tailored action.

WHO's Global status report on neurology,^14^ published in 2025, provides a comprehensive picture of the current gaps and needs of WHO Member States, providing a baseline from which to monitor progress towards achieving the ten global targets set out in IGAP. The intention of the report is that countries can build targeted, integrated and sustainable responses to neurological disorders. Data were collected from ministries of health focal points of 102 countries (53% of the world's countries, representing 71% of the global population). The report emphasizes the collective action needed to strengthen the response to neurological disorders to meet the targets of IGAP by 2031.

These signs of policy prioritization are positive steps towards addressing PD on a global scale and through a public health perspective. However, they must also be met with implementation at the country level. Attention and interest in PD are certainly growing and have been met with an increase in major funding in the PD research space, for example, from The Michael J. Fox Foundation for Parkinson's Research (MJFF), the world's largest non-profit funder of PD research. Engaging a more diverse pool of funders moving forward would ensure larger scale research and implementation and sustainability of initiatives, while sharing the burden of progress. However, without the appropriate allocation of resources, monitoring and evaluation at the country level, sustainable progress will not be realized. Investment and buy-in by national governments are crucial to ensure sustained, collaborative and multi-sectoral action to meet the objectives of IGAP. How interventions are implemented will depend on the country's context, availability of resources and needs of the population. Implementation will require a whole-society approach, with input from policymakers, program managers, service planners, health practitioners, civil society organizations, professional societies, advocacy groups, academic institutions, donors and funders.^13^ People living with neurological disorders, their carers and families should also be at the heart of action.

## The power of partnerships in Parkinson's disease policy and research

A global and unified response to PD also requires a shift towards more integrated and systems-oriented approaches. One key opportunity lies in aligning PD efforts with broader initiatives targeting dementia and other neurological disorders, mental health, and non-communicable diseases, particularly considering the rising prevalence of non-communicable disease in LMICs and similarities in chronic care needs. One example is WHO's iSupport for Dementia,^15^ a training and support manual for carers of people with dementia, which can be adopted for carers of persons with PD. This tool is currently being adapted for care partners of people with PD and cognitive impairment.^16^

Furthermore, health systems in many LMICs are not structured around highly specialized services, such as memory clinics commonly seen in HICs, meaning that single-disease approaches, while valuable, may be insufficient to achieve rapid and large-scale impact. This is especially evident in efforts to improve access to medicines and care, where system-wide barriers, such as procurement, workforce capacity and stigma, affect multiple conditions.^17^ Approaches such as those outlined in the WHO's ‘fishbone diagram’ on access to medicines for neurological disorders propose the utilization of ‘tracer conditions’ – specific diseases used to understand wider issues across disease groups – to offer pragmatic pathways to strengthening access and care more widely by addressing and strengthening health system components.^17^

Further learning for addressing PD can be drawn from other disease initiatives, such as the global response to HIV and AIDS,^18^ where fear, stigma and limited scientific understanding catalyzed substantial investment in research, infrastructure and advocacy. Although PD is not infectious, it is associated with significant and multi-level stigma^19^ and a growing burden,^2,3^ underscoring the urgent need for increased investment in research and health system capacity. However, PD, and other age-associated conditions such as dementia, have not historically been prioritized within global health agendas. The inaction in policy and funding is largely due to their chronic, progressive nature, lower immediate mortality, long-standing absence of disease-modifying therapies and ageism towards the health issues of older people – particularly where resources are constrained. The global response to HIV and AIDS illustrates that strategic framing, accountability mechanisms and sustained advocacy can mobilize global action for PD, and other neurological disorders. As cited in the WHO's ‘Six action steps’, key actions must be coordinated to generate strategies, programs and services, and PD must be prioritized on policy agendas.^11^

Increased global investment in research on PD is essential to inform prevention, diagnosis, treatment and care, though is marred by inequities in the allocation of funding and volume of outputs across World Bank income groups. HICs, unsurprisingly, lead the way in the availability of research funding and generation of data,^14,20–24^ though diversity in PD research and access to care in HICs also remains a significant challenge.^25–30^ The development of priority research agendas is crucial to ensure impact-driven research by multisectoral collaborators and partnerships that address diversity and equity and involve persons with lived experience and policymakers from the outset.

One such example of a multi-country, multi-method research project is ‘Transforming Parkinson's Care in Africa’ (TraPCAf), a UK-funded National Institute for Health and Care Research (NIHR) Global Health Research Group.^31^ The project is addressing the significant gap in the availability of data across Africa, seeking to develop novel insights to address risk reduction, diagnostic, treatment and care pathways and a policy response in six African countries. Data include (1) the epidemiological and economic burden, (2) environmental, genetic and gut-related risk factors,^32^ (3) the utility of novel diagnostic methods (e.g., the use of a sebum swab inspired by Joy Milne, the ‘super smeller’^33^), (4) the lived experience of people with PD and carers, (5) a clinical trial of *Mucuna Pruriens*^34,35^ and (6) the clinical phenotype among diverse African populations. Such evidence will offer important insights to improve the public health response to PD across the continent.

Another example of the power of partnerships and collaboration in research is the Global Parkinson's Genetics Program (GP2),^36^ which plays a central role in convening global research groups to harmonize large scale genetic and clinical data (250,000 individuals) to drive forward the PD research agenda. Through the prioritization of under-represented populations, GP2 is expanding the global evidence base, particularly in LMICs, while investing in training, capacity building and infrastructure development.^24^ GP2 is a resource program of Aligning Science Across Parkinson's (ASAP), a coordinated research initiative designed to accelerate PD discoveries through collaboration, research-enabling resources, and data sharing.^37^ ASAP launched in 2019 and currently supports GP2, the Collaborative Research Network (CRN)^38^ and Parkinson's Progression Markers Initiative (PPMI),^39^ all of which are implemented by MJFF.^40^

Such examples align with policy calls to provide appropriate funding to conduct and implement research, and build research capacity where needed, to generate high-quality data on PD.^11^ WHO's Global status report on neurology shows that just 29% of countries reported routinely collecting core indicators for PD and integrating them into the health information system, while 31% of countries compile and report data on PD. Integrating research data with national health system indicators is crucial in the generation of a public health response to PD. Integration will require strong partnerships between researchers, ministries, civil society and WHO to align evidence with routine metrics, e.g., workforce capacity and service access.

## Global Parkinson's disease advocacy efforts

IGAP acknowledges advocacy as the first step in raising awareness and promoting a better understanding of neurological disorders at all levels of society.^12^ Effective advocacy – including through public awareness campaigns – can influence policy, practice and attitudes, including the mobilization of resources to support policy prioritization. IGAP global target 1.2 states that 100% of countries will have at least one functioning awareness campaign or advocacy program for neurological disorders by 2031. WHO's Global status report on neurology^14^ reported just 33% of responding countries had an awareness raising campaign or advocacy program for PD as of 2022, suggesting more needs to be done to raise the profile of the condition globally.

Global advocacy efforts relating to PD have accelerated in recent years, shifting from awareness-raising and community support to broader policy engagement, advocacy for structural change and the prioritization of PD in national health plans and budgets. A notable example is the National Plan to End Parkinson's Act (2024),^41^ the first United States of America legislation to develop and implement a comprehensive national strategy on PD, i.e., a ‘National Parkinson's Project’ within the department of Health and Human Services.

Global advocacy and awareness efforts often center around ‘World Parkinson's Day’ on April 11^th^, and to a lesser extent on World Movement Disorders Day (November 29^th^) and World Brain Day (July 22^nd^). Over the past few years, this has involved a united global response through ‘Spark the Night’, an annual awareness campaign on April 11^th^ to recognize and unite the global community through lighting up buildings and other landmarks in blue. In 2025, the campaign featured 500 sites in more than 200 cities, though included just two locations in LMICs (Ghana and Kenya).^42^

‘Spark the Night’ is spearheaded by the PD Avengers, an organization founded in 2020 as a ‘Global Alliance to End Parkinson's’. PD Avengers is a patient-led movement with the aim of uniting people with PD, care partners, clinicians, researchers, and allied organizations to accelerate action globally. The organization's philosophy is ‘think global, act local’, combining a broad global vision across three pillars – wellness, advocacy and research – with community-level action. PD Avengers is well positioned to act as a global advocacy catalyst by uniting stakeholders to demand policy action, to raise awareness, improve access to care, and shift the pace of scientific discovery. At the time of publication, the PD Avengers membership spans 106 countries and territories, including many LMICs across different world regions. The elevation of experts by lived experience, i.e., a commitment to the deliberate positioning of people affected by PD as authoritative voices in public discourse, research agenda setting, and policy formation within global PD advocacy, has been an important shift over the past decade, and particularly since the Covid-19 pandemic when virtual connection became the norm. PD Avengers is an excellent example of this new model of grassroots, experience-led advocacy.

Paving the way in patient advocacy since 2006, however, has been the World Parkinson Congress (WPC).^43^ The World Parkinson Congress is a triennial conference organized by the World Parkinson Coalition (founded by world-renowned neurologist Dr Stanley Fahn), that involves and centers around the inclusion of people living with PD and care partners, alongside scientists and clinicians. The Congress features scientific, wellness and creative programs, including art, film, dance and music to create an inclusive, informative and uplifting experience. The centering of lived experience and action towards a rights-based, person-centered approach to advocacy by the global PD community reflects the operationalization of WHO's framework for meaningful engagement of people living with non-communicable diseases, mental health and neurological conditions to co-create and enhance policies, programs and services.^44^ It aligns with IGAP's guiding principles on the engagement and empowerment of people with neurological conditions and caregivers across planning and service delivery, in policy and legislation development, program implementation, advocacy, research, monitoring and evaluation.^12^

Regional and country PD non-profit organizations play a crucial role as bodies dedicated to advocacy, support, awareness, research and health policy, though they vary in scope, scale and structure. Such organizations across HICs are well-established and developed, for example, Parkinson's Europe (previously the European Parkinson's Disease Association) founded in 1992.^45^ However, regional and local infrastructure is developing across many LMICs. An example of such a network is Parkinson's Africa.^46^ Registered in 2021, the pan-African charity is dedicated to supporting and empowering African's affected by PD through working directly with partner organizations, program coordinators, trained community leaders, people impacted by PD, and health professionals across Africa.

The International Parkinson and Movement Disorder Society (MDS), founded in 1985 by Dr Stanley Fahn and Dr C. David Marsden, is another example of a professional society dedicated to advancing the field of movement disorders.^47^ MDS serves as a global voice of over 12,000 professionals, as a catalyst to advance research, and as an essential source of education and knowledge dissemination.^48^ The society continues to expand its geographical reach and, in 2026 in collaboration with GP2, held the first MDS African Parkinson's Disease and Movement Disorder Conference in Africa.

Despite the progress made in global advocacy and awareness, inequalities in access to care, treatment and support persist, while LMIC and under-represented populations remain largely excluded from research.^20^ Disparities span the research continuum, from epidemiology to clinical trials, compounded by structural limitations, including resource constraints. The unique challenges of countries, communities and minoritized groups require tailored and appropriate strategies that span the socioecological spectrum, i.e., from the individual (e.g., environmental risk) to global (e.g., brain health policy).^49^ In order to address these inequities, the inaugural World Parkinson's Summit – co-sponsored by the Parkinson's Foundation (USA) and the Fresco Parkinson Institute (Italy) – was held in 2025.^49^ The meeting, involving 26 attendees from 11 countries across five continents and six international health-related organizations and foundations, sought to develop a global approach to PD care, with a particular focus on how multi-national organizations can better support people affected by PD. Key priority areas identified during the Summit include action to generate better data (including development of registries and quantification of cost of care gaps), the development of actionable campaigns, creation of global care standards, launch of access pilots, training of the primary care workforce, and development of ethical digital health and artificial intelligence tools.^49^

Advocacy and awareness initiatives are also increasingly embedded into research programs globally, with a particular focus on community engagement and participatory approaches to research. The adoption of participatory approaches and tailored, culturally sensitive engagement not only improves community awareness of PD but can also lead to more inclusive approaches to involving marginalized communities in research.^27,29^ An example of a successful approach featured as an ‘IGAP in action’ case study in the Global status report on neurology^14^ is ‘Transforming Parkinson's Care in Africa’ (TraPCAf), which has embedded community engagement strategies across Africa.^31^ Strategies have included patient advisory groups to inform and feedback on tools and recruitment procedures, the establishment of seven patient support groups across six countries to ensure continued support for the cohort, and community-awareness raising initiatives using culturally sensitive approaches.

Another example is the ‘Latin American Research Consortium on the Genetics of Parkinson's Disease’ (LARGE-PD).^50^ Established in 2006, this 13-country initiative across 37 sites aims to advance genetic research and community engagement across the region, involving educational outreach to overcome barriers such as limited awareness and language differences, the development of multilingual educational materials, and patient-partnership models to ensure research addresses community needs. Since 2024, LARGE-PD has collaborated with Parkinson's Foundation's PD GENEration in Latin America to expand its offering of genetic testing and counselling to nine additional countries in the region,^51^ increasing the engagement reach of LARGE-PD, who recently reached the milestone of recruiting 10,000 participants.

## Policy, advocacy and research recommendations

Achieving a unified global PD response will require sustained, multi-sectoral, concerted action. The WHO's IGAP toolkit^13^ offers an important opportunity to develop policy, advocacy and research priorities, centering on five cross-cutting strategic objectives: prioritization and governance; diagnosis, treatment and care; promotion and prevention; research and information systems; and a PD-specific approach (Table 1).

**Table 1.** Recommendations for a unified global response to Parkinson's disease, adapted from WHO's IGAP strategic objectives for an integrated approach to neurological disorders.^13^

Strategic objective	Recommendations
Prioritization and governance	Build global advocacy and awareness. Develop and implement evidence-based policy, plans and legislation. Strengthen health systems financing.
Diagnosis, treatment and care	Strengthen holistic, multi-level care pathways. Build capacity of the health workforce. Improve access to medicines and technologies. Strengthen carer support.
Promotion and prevention	Promote general brain health. Promote prevention and risk reduction strategies.
Research and information systems	Improve research investment and output. Develop health information systems.
A PD-specific approach	Advocate for PD-specific policy, advocacy and research.

### Prioritization and governance

Prioritization and governance will require advocacy and awareness raising at the global and local level, building on initiatives and opportunities to mobilize stakeholders to catalyze change. Proposed actions include effective stakeholder engagement to develop PD-specific advocacy agendas, strategies and awareness raising campaigns. Such efforts may also align with broader health efforts, for example, country-level responses to non-communicable diseases and mental health. Prioritization of PD will also be dependent on the implementation of policies, plans and legislation which signal political commitment. PD should be integrated and mainstreamed into national policy as a starting point for effective allocation of resources and configuration of health systems to address the growing burden posed by the condition. Appropriate health system financing and budget allocation for PD needs to be coupled with an assessment of burden and generation of an investment case. Understanding the economic, health and social benefits of investing in PD will generate persuasive arguments for prioritization.

### Diagnosis, treatment and care

Timely and appropriate diagnosis and treatment is paramount for PD, though challenges with the accessibility of services, specialists and medicines persist globally.^17,30,52–55^ Access to holistic care is unevenly distributed across the world, and within countries, with urban-rural and socioeconomic disparities. Integrated, person-centered and responsive care that meets the needs of all populations is needed, with a shift to a focus on primary and community care. The development and implementation of training models, such as through WHO's mental health Gap Action Programme Intervention Guide (mhGAP-IG)^56^ would be beneficial for PD. However, building the specialist neurological workforce is also paramount, particularly among LMICs and in rural regions – the total neurological workforce is just 0.1 per 100,000 in low-income countries, compared to 7.1 per 100,000 in HICs.^57^ MDS continue to make significant strides in upskilling neurologists, physicians and allied health professionals globally through regional courses, e-learning, outreach education, train the trainer programs and center to center training.^58^ Similarly, the Edmond J. Safra Fellowship in Movement Disorders is expanding its reach to LMICs^59^ – the Edmond J. Safra Foundation is another major funder of PD research and capacity building.^60^

Improvements in diagnosis must also be met with improvements in the availability and affordability of PD medicines. In most LMICs, and among marginalized populations in many HICs, patients’ functioning and quality of life are degraded by the lack of access to treatments for levodopa-related motor response complications, or indeed to levodopa/carbidopa more generally.^17,18,61^ Advancements in the availability of treatments – including device-aided therapies, such as deep brain stimulation or continuous infusion therapies – remain limited to high-income regions of the world.^52^ Improvements in medicines access are complex, though will stem from improved policy prioritization.^17^ Finally, the provision of support for carers is vital as the burden of informal caregiving has profound social, physical and financial risks.^62,63^

### Promotion and prevention

Promotion of brain health and prevention of PD requires an intersectoral approach that stems from the prioritization of population-wide neurological health strategies. Though we are increasingly learning more about the causes of, and contributing factors to, PD, prevention is more challenging at the individual level, in that the causes of PD remain uncertain. The evidence base for environmental exposures is growing^64,65^ while we continue to learn more about the role of genetics in PD etiology,^66,67^ including in underserved populations, for example, the recently identified novel GBA1 gene variant in persons of African ancestry identified in a Nigerian cohort.^68^

Most PD cases will likely result from a complex interplay of ageing, environmental, genetic, and epigenetic factors. In the future, subtyping of PD and the generation of polygenic risk scores may support the development of precision medicine.^69^ However, for now, general health messaging around exercise, diet and exposure to environmental risk factors (e.g., pesticides) are important – such messaging should be communicated through advocacy efforts globally. One such strategy emphasizes the ‘Parkinson PLAN’ to address the ‘Parkinson pandemic’,^70,71^ with a focus on preventing new cases, learning why PD starts and progresses, amplifying the voices of people with PD, and navigating the frontier of new treatments.

### Research and information systems

Improved investment in research is key to enabling a better and holistic understanding of PD. Research should be multidisciplinary, multi-method, collaborative and should promote capacity building and the development of infrastructure. An example of such development is the commissioning of the GP2-College of Medicine University of Lagos Molecular Laboratory for Parkinson's Disease Research, the first dedicated molecular facility of its kind in Nigeria which will enable local analysis of genetic data and situate West Africa as a generator of scientific progress in PD research.^72^ The generation of evidence is key to informing policy – such evidence should also come from health information systems, including electronic health records and disease registries. Efforts must be made to improve the inclusivity of research to overcome the under-representation of certain populations in our current understanding of PD. Involving and engaging persons with lived experience throughout the research cycle is also paramount and will ensure that research outcomes meet the needs of the populations intended to benefit.

### A PD-specific approach

While many opportunities and recommendations for policy, advocacy and research are cross-cutting and integrated, it remains crucial to address PD as an individual neurological condition. PD has unique biological mechanisms, symptoms and progression pathways, resulting in specific lived experiences and needs. Further, in a competitive funding landscape, recognition of PD and its unique and significant burden provides a stronger case for dedicated funding, investment, advocacy and prioritization. Having said that, improving services for one condition leads to advances in management and care across neurological disorders. Synergistic action, with consideration for constrained resources and competing priorities, will produce the greatest return.

## Conclusion

The global policy and advocacy response to PD is slowly, but surely, beginning to align with the growing physical, social and economic burden the condition poses worldwide. Significant strides have been made with regard to the promotion of PD as a public health priority, which must now be met by local action and continued investment by funders. There are excellent examples of the power and importance of partnerships and collaboration, particularly in the research and advocacy space. Such partnerships must now extend more broadly to ensure that no one with PD is left behind, and continue to involve governments, non-governmental organizations and private sectors to ensure sustained and multi-level action. Achieving a unified global response to PD should center on the five cross-cutting objectives outlined in WHO's IGAP toolkit.^13^

## References

[1] Steinmetz JD Seeher KM Schiess N , et al. Global, regional, and national burden of disorders affecting the nervous system, 1990–2021: a systematic analysis for the Global Burden of Disease Study 2021. Lancet Neurol 2024; 23: 344–381. 38493795 10.1016/S1474-4422(24)00038-3 PMC10949203

[2] Li M Ye X Huang Z , et al. Global burden of Parkinson's disease from 1990 to 2021: a population-based study. BMJ Open 2025; 15: e095610–20250427. 10.1136/bmjopen-2024-095610 PMC12035419 40288800

[3] Su D Cui Y He C , et al. Projections for prevalence of Parkinson’s disease and its driving factors in 195 countries and territories to 2050: modelling study of Global Burden of Disease Study 2021. Br Med J 2025; 388: e080952. 10.1136/bmj-2024-080952 PMC11881235 40044233

[4] Wang S Che Y Lin Y , et al. Epidemiology of Parkinson’s disease – global burden of disease research from 1990 to 2021 and future trend predictions. Clin Parkinsonism Relat Disord 2026; 14: 100421. 10.1016/j.prdoa.2026.100421 PMC12828836 41584999

[5] Peng S Liu P Wang X , et al. Global, regional and national burden of Parkinson’s disease in people over 55 years of age: a systematic analysis of the global burden of disease study, 1991–2021. BMC Neurol 2025; 25: 78. 40269818 10.1186/s12883-025-04191-8 PMC12016353

[6] Pereira GM Teixeira-dos-Santos D Soares NM , et al. A systematic review and meta-analysis of the prevalence of Parkinson’s disease in lower to upper-middle-income countries. NPJ Parkinsons Dis 2024; 10: 81. 39349513 10.1038/s41531-024-00779-y PMC11442769

[7] Gandhi SE Grosset KA Iruthayaraj PA , et al. Comparative analysis of the incidence, prevalence, and survival of 8 types of parkinsonism in a population-based study with 367 million person years of observation over 21 years. Mov Disord Clin Pract 2026; 13: 933–948. 41125074 10.1002/mdc3.70368 PMC13071333

[8] Aamodt WW Willis AW Dahodwala N . Racial and ethnic disparities in Parkinson disease: a call to action. Neurol Clin Pract 2023; 13: e200138. 20230316. 10.1212/CPJ.0000000000200138 PMC10101714 37064587

[9] Dorsey ER Elbaz A Nichols E , et al. Global, regional, and national burden of Parkinson's disease, 1990-2016: a systematic analysis for the Global Burden of Disease Study 2016. Lancet Neurol 2018; 17: 939–953. 30287051 10.1016/S1474-4422(18)30295-3 PMC6191528

[10] World Health Organization. Parkinson disease: a public health approach. Technical Brief. Geneva: World Health Organization, 2022. https://www.who.int/publications/i/item/9789240050983

[11] Schiess N Cataldi R Okun MS , et al. Six action steps to address global disparities in Parkinson disease: A World Health Organization priority. JAMA Neurol 2022. DOI: 10.1001/jamaneurol.2022.1783 35816299

[12] World Health Organization. Intersectoral global action plan on epilepsy and other neurological disorders 2022–2031. Geneva: World Health Organization, 2023. https://www.who.int/publications/i/item/9789240076624

[13] World Health Organization. Intersectoral global action plan on epilepsy and other neurological disorders 2022–2031: implementation toolkit. Geneva: World Health Organization, 2024. https://www.who.int/publications/i/item/9789240096356 10.1227/neu.0000000000002828 38224233

[14] World Health Organization. Global status report on neurology. Geneva: World Health Organization, 2025. https://www.who.int/publications/i/item/9789240116139

[15] World Health Organization. iSupport for Dementia. Geneva: World Health Organization, 2019. https://www.who.int/publications/i/item/9789241515863

[16] Hand A Samarakoon D Greenwell K , et al. Development and feasibility trial of iSupport-PD, a digital intervention for carers of people with Parkinson’s and cognitive impairment. *ISRCTN* 2025. 10.1186/ISRCTN16483203

[17] World Health Organization. Improving access to medicines for neurological disorders. Geneva: World Health Organization, 2024. https://iris.who.int/handle/10665/378216

[18] Subramanian I Okubadejo N Fothergill-Misbah N . Levodopa—an oldie but a goodie—time to make it accessible to all in Sub-Saharan Africa. Mov Disord 2025; 40: 1034–1036. 40189922 10.1002/mds.30180

[19] Crooks S Mitchell G Wynne L , et al. Exploring the stigma experienced by people affected by Parkinson’s disease: a systematic review. BMC Public Health 2025; 25: 25. 39754132 10.1186/s12889-024-21236-8 PMC11697948

[20] Teixeira-dos-Santos D Tan A-H Okubadejo N , et al. Bridging global diversity gaps in Parkinson disease research. Nat Rev Neurol 2026. DOI: 10.1038/s41582-026-01183-1 41667846

[21] Lim S-Y Tan AH Ahmad-Annuar A , et al. Uncovering the genetic basis of Parkinson's disease globally: from discoveries to the clinic. Lancet Neurol 2024; 23: 1267–1280. 39447588 10.1016/S1474-4422(24)00378-8

[22] Tan AH Cornejo-Olivas M Okubadejo N , et al. Genetic testing for Parkinson's disease and movement disorders in less privileged areas: barriers and opportunities. Mov Disord Clin Pract 2024; 11: 14–20. 38291851 10.1002/mdc3.13903 PMC10828609

[23] Schumacher-Schuh AF Bieger A Okunoye O , et al. Underrepresented populations in Parkinson's genetics research: current landscape and future directions. Mov Disord 2022; 37: 1593–1604. 35867623 10.1002/mds.29126 PMC10360137

[24] Step K Eltaraifee E Elsayed I , et al. Advancing Parkinson's disease research in Africa: a strategic training framework of the global Parkinson's genetics program. Mov Disord 2025; 40: 51–56. 39482233 10.1002/mds.30051 PMC11752974

[25] Subramanian I Mathur S Oosterbaan A , et al. Unmet needs of women living with Parkinson's disease: gaps and controversies. Mov Disord 2022; 37: 444–455. 35060180 10.1002/mds.28921

[26] Ison JM Jackson JD Hemley H , et al. Fostering inclusivity in research engagement for underrepresented populations in Parkinson's disease: the FIRE-UP PD study. Contemp Clin Trials 2024; 144: 107619. 38971301 10.1016/j.cct.2024.107619

[27] Ramadhan M Schrag A Tufail M , et al. Participation of ethnic minorities in Parkinson’s research: challenges and needs. A qualitative study. Age Ageing 2025: 54. DOI: 10.1093/ageing/afaf296 PMC12551381 41134531

[28] Siddiqi S Ortiz Z Simard S , et al. Race and ethnicity matter! Moving Parkinson’s risk research towards diversity and inclusiveness. NPJ Parkinsons Dis 2025; 11: 45. 40050644 10.1038/s41531-025-00891-7 PMC11885646

[29] Dobreva I Kalam S Roche M , et al. Understanding barriers and facilitators to participation in Parkinson's research in Black communities in the UK. J Parkinsons Dis 2025; 15: 1035–1039. 20250620. 40538322 10.1177/1877718X251351990 PMC12374798

[30] Ramadhan M Stott J Schrag A . The barriers to receiving health care for people with Parkinson’s from predominantly Asian backgrounds in the UK. NPJ Parkinsons Dis 2025; 11: 131. 40394050 10.1038/s41531-025-00946-9 PMC12092625

[31] Walker R Fothergill-Misbah N Kariuki S , et al. Transforming Parkinson's care in Africa (TraPCAf): protocol for a multimethodology National Institute for Health and Care Research Global Health Research Group project. BMC Neurol 2023; 23: 73. 37858118 10.1186/s12883-023-03414-0 PMC10585779

[32] Okubadejo NU Schaeffer E Noyce AJ , et al. Addressing gaps in Parkinson's disease etiology: The need for a polyexposure score. Mov Disord 2026. Early View. 10.1002/mds.70231 41704037

[33] Sarkar D Sinclair E Lim SH , et al. Paper spray ionization Ion mobility mass spectrometry of sebum classifies biomarker classes for the diagnosis of Parkinson’s disease. JACS Au 2022; 2: 2013–2022. 36186554 10.1021/jacsau.2c00300 PMC9516698

[34] Fothergill-Misbah N Maroo H Cham M , et al. Could Mucuna pruriens be the answer to Parkinson's disease management in sub-Saharan Africa and other low-income countries worldwide? Parkinsonism Relat Disord 2020; 73: 3–7. 32179240 10.1016/j.parkreldis.2020.03.002

[35] Hammoud F Ismail A Zaher R , et al. Mucuna pruriens treatment for Parkinson disease: a systematic review of clinical trials. Parkinsons Dis 2025; 2025: 1319419. 20250818. 40860042 10.1155/padi/1319419 PMC12377966

[36] Singleton A Blauwendraat C Morris HR , et al. Parkinson's disease: emerging opportunities through global collaboration. Lancet 2026; 407: 203–205. 41077048 10.1016/S0140-6736(25)01910-5

[37] ASAP. Uncovering the roots of Parkinson's disease, https://parkinsonsroadmap.org/ (2026, accessed 14 April 2026).

[38] CRN. Sparking new discoveries for Parkinson's disease, https://www.asapcrn.org/ (2026, accessed 14 April 2026).

[39] PPMI. About PPMI, https://www.ppmi-info.org/about-ppmi (2026, accessed 14 April 2026).

[40] MJFF. ASAP initiative, https://www.michaeljfox.org/asap (2026, accessed 14 April 2026).

[41] U.S. Congress. H.R. 2365, Dr. Emmanuel Bilirakis and Honorable Jennifer Wexton National Plan to End Parkinson’s Act, https://www.congress.gov/bill/118th-congress/house-bill/2365 (2023).

[42] Spark The Night. Top landmarks around the world to spark blue for Parkinson’s awareness on April 11, https://www.worldparkinsonsnight.com/2025-media-release (2025, accessed 03 March 2026).

[43] World Parkinson Congress. 7th World Parkinson Congress, https://wpc2026.org/ (2026, accessed 06 March 2026).

[44] World Health Organization. WHO framework for meaningful engagement of people living with noncommunicable diseases, and mental health and neurological conditions. Geneva: World Health Organization, 2023. https://www.who.int/publications/i/item/9789240073074

[45] Parkinson's Europe. Parkinson's Europe: Who we are, https://parkinsonseurope.org/who-we-are/ (2026, accessed 06 March 2026).

[46] Parkinson's Africa. Parkinson's Africa: What we do, https://www.parkinsonsafrica.org/about-us/what-we-do/ (2026, accessed 06 March 2026).

[47] MDS. About the Society, https://www.movementdisorders.org/MDS/What-We-Do.htm (2026, accessed 14 April 2026).

[48] MDS. MDS Purpose, Mission & Goals, https://www.movementdisorders.org/MDS/About/Who-We-Are/Purpose-Mission–Goals.htm (2026, accessed 14 April 2026).

[49] Mantri S Ghilardi MF Lessard N , et al. Proceedings of the world summit on Parkinson’s disease. NPJ Parkinsons Dis 2025; 11: 293. 41087391 10.1038/s41531-025-01123-8 PMC12521725

[50] Zabetian CP Mata IF ; PD obotLARCotGo. LARGE-PD: examining the genetics of Parkinson's disease in Latin America. Mov Disord 2017; 32: 1330–1331. 28657124 10.1002/mds.27081

[51] Parkinson's Foundation. Parkinson’s Foundation Genetics Study to Launch in 10 Latin American Countries, https://www.parkinson.org/blog/research/pd-gene-latam-expansion (2025, accessed 03 March 2026).

[52] Goh ZHK Cheong JLY Marras C , et al. Surveying global availability of Parkinson’s disease treatment. J Parkinsons Dis 2022; 12: 1023–1034. 35147549 10.3233/JPD-213006

[53] Hamid E Ayele BA Massi DG , et al. Availability of therapies and services for Parkinson's disease in Africa: a continent-wide survey. Mov Disord 2021; 36: 1393–2407. 10.1002/mds.28669 34080713

[54] Mohamed Ibrahim N Phuenpathom W Jagota P , et al. Practices, resources and challenges in Parkinson's disease management in Asia: movement disorders in Asia study group report. Mov Disord Clin Pract 2026; 13: 379–392. 41299861 10.1002/mdc3.70447 PMC12911452

[55] Jeyachandran C Spooner C Salgado AMR , et al. Barriers and facilitators to accessing healthcare for people with Parkinson's disease in Latin America: a qualitative study. Health Expect 2025; 28: e70380. 10.1111/hex.70380 PMC12344579 40801231

[56] World Health Organization. mhGAP intervention guide for mental, neurological and substance use disorders in non-specialized health settings: mental health Gap Action Programme (mhGAP) – version 2.0. Geneva: World Health Organization, 2016. https://www.who.int/publications/i/item/9789241549790 27786430

[57] World Health Organization. Atlas: country resources for neurological disorders - 2nd ed. Geneva: World Health Organization, 2017. https://www.who.int/publications/i/item/atlas-country-resources-for-neurological-disorders

[58] MDS. Education, https://www.movementdisorders.org/MDS/Education.htm (2026, accessed 14 April 2026).

[59] MJFF. The Edmond J. Safra Fellowship in movement disorders, https://www.michaeljfox.org/edmond-j-safra-fellowship-movement-disorders (2026, accessed 14 April 2026).

[60] Edmond J. Safra Philanthropic Foundation. Michael J. Fox Foundation for Parkinson's Research, https://www.edmondjsafra.org/2022/07/29/michael-j-fox-foundation-for-parkinsons-research/ (2026 , accessed 14 April 2026).

[61] Fothergill-Misbah N Cataldi R Stothard M , et al. Parkinson disease treatments on national essential medicines lists, African region. Bull World Health Organ 2025; 103: 626–634. 20250825. 41035551 10.2471/BLT.25.293460 PMC12481222

[62] Sin WWF Chan LML Ng CTY , et al. Factors impacting caregiver burden in Parkinson's disease: a systematic review and meta-analysis. Int J Nurs Stud 2026; 174: 105299. 41330214 10.1016/j.ijnurstu.2025.105299

[63] Hulshoff M Sun C Book E , et al. Care partner needs in Parkinson's disease: a systematic review of qualitative and quantitative data. Journal of Parkinson’s Disease 2025; 15: 1043–1061. 10.1177/1877718X251344066 PMC13347510 40448327

[64] Dorsey ER Bloem BR . Parkinson’s disease is predominantly an environmental disease. Journal of Parkinson’s Disease 2024; 14: 451–465. 10.3233/JPD-230357 PMC11091623 38217613

[65] Dorsey ER De Miranda BR Hussain S , et al. Environmental toxicants and Parkinson's disease: recent evidence, risks, and prevention opportunities. Lancet Neurol 2025; 24: 976–986. 41109237 10.1016/S1474-4422(25)00287-X

[66] Lim S-Y Klein C . Parkinson’s disease is predominantly a genetic disease. Journal of Parkinson’s Disease 2024; 14: 467–482. 10.3233/JPD-230376 PMC11091652 38552119

[67] Blauwendraat C Nalls MA Singleton AB . The genetic architecture of Parkinson's disease. Lancet Neurol 2020; 19: 170–178. 31521533 10.1016/S1474-4422(19)30287-X PMC8972299

[68] Rizig M Bandres-Ciga S Makarious MB , et al. Identification of genetic risk loci and causal insights associated with Parkinson's disease in African and African admixed populations: a genome-wide association study. Lancet Neurol 2023; 22: 1015–1025. 37633302 10.1016/S1474-4422(23)00283-1 PMC10593199

[69] Marras C Fereshtehnejad S-M Berg D , et al. Transitioning from subtyping to precision medicine in Parkinson's disease: a purpose-driven approach. Mov Disord 2024; 39: 462–471. 38243775 10.1002/mds.29708

[70] Dorsey ER Okun MS Bloem BR . A PLAN to address the Parkinson pandemic. Journal of Parkinson’s Disease 2025; 15: 1322–1336. 10.1177/1877718X251378115 PMC13347541 41117498

[71] Dorsey ER Bloem BR . The Parkinson pandemic-A call to action. JAMA Neurol 2018; 75: 9–10. 2017/11/14. 29131880 10.1001/jamaneurol.2017.3299

[72] GP2. GP2 Visits Nigeria: Commissioning of Nigeria's First Molecular Laboratory for Parkinson's Disease Genetics, https://gp2.org/gp2-visits-nigeria-commissioning-of-nigerias-first-molecular-laboratory-for-parkinsons-disease-genetics/ (2026, accessed 14 April 2026).

